# Buprenorphine

**DOI:** 10.1107/S1600536814009672

**Published:** 2014-05-03

**Authors:** Jaroslaw Mazurek, Marcel Hoffmann, Anna Fernandez Casares, Phillip D. Cox, Mathew D. Minardi

**Affiliations:** a Crystallics B.V., Meibergdreef 31, 1105 AZ Amsterdam, The Netherlands; b Noramco Inc., 503 Carr Rd, Suite 200, Wilmington, DE 19809, USA; c Noramco Inc., 1440 Olympic Drive, Athens, GA 30601, USA

## Abstract

In the crystal structure of a semi-synthetic opioid drug buprenorphine, C_29_H_41_NO_4_ {systematic name: (2*S*)-2-[(5*R*,6*R*,7*R*,14*S*)-9α-cyclo­propyl­methyl-3-hy­droxy-6-meth­oxy-4,5-ep­oxy-6,14-ethano­morphinan-7-yl]-3,3-di­methyl­butan-2-ol}, the cyclo­propyl­methyl group is disordered over two sites with an occupancy factor of 0.611 (3) for the major component. One of the hy­droxy groups is involved in intra­molecular O—H⋯O hydrogen bond. The other hy­droxy group acts as a proton donor in an inter­molecular O—H⋯O inter­action that connects mol­ecules into a zigzag chain along the *b* axis.

## Related literature   

For the crystal structure of buprenorphine hydro­chloride, see: Flippen-Anderson *et al.* (1994 ▶); Kratochvil *et al.* (1994 ▶). For pharmacological information on buprenorphine, see: Weinberg *et al.* (1988 ▶); Huang *et al.* (2001 ▶). For the Kitaigorodskii packing coefficient, see: Kitajgorodskij (1973 ▶).
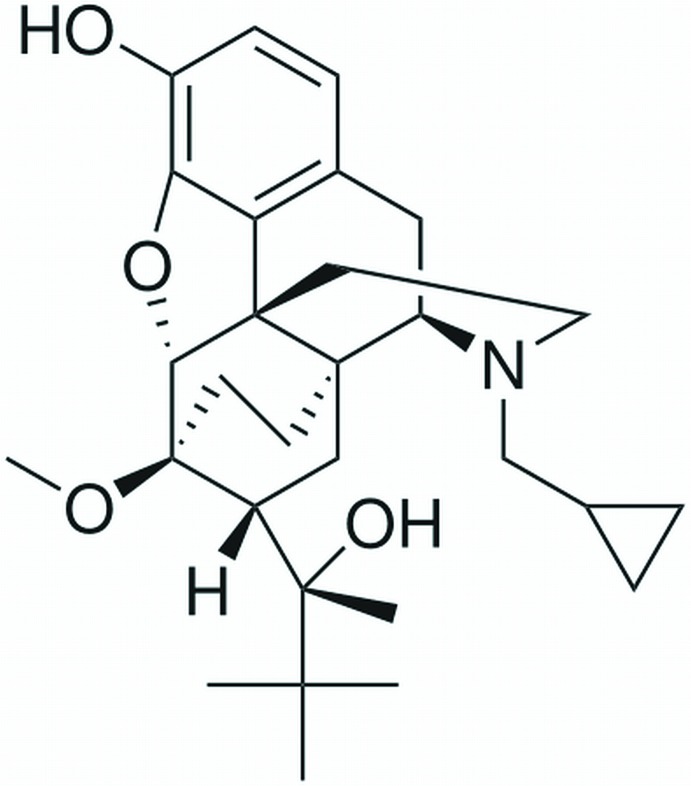


## Experimental   

### 

#### Crystal data  

C_29_H_41_NO_4_
*M* *_r_* = 467.63
Monoclinic, 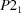

*a* = 9.8154 (6) Å
*b* = 10.4283 (9) Å
*c* = 13.4508 (9) Å
β = 108.796 (5)°
*V* = 1303.37 (16) Å^3^
*Z* = 2
Mo *K*α radiation
μ = 0.08 mm^−1^
*T* = 296 K
0.45 × 0.45 × 0.25 mm

#### Data collection  

Bruker KappaCCD diffractometer
14483 measured reflections
4886 independent reflections
4312 reflections with *I* > 2σ(*I*)
*R*_int_ = 0.026

#### Refinement  

*R*[*F* ^2^ > 2σ(*F* ^2^)] = 0.052
*wR*(*F* ^2^) = 0.146
*S* = 1.04
4886 reflections
352 parameters
112 restraints
H atoms treated by a mixture of independent and constrained refinement
Δρ_max_ = 0.32 e Å^−3^
Δρ_min_ = −0.31 e Å^−3^

### 

Data collection: *COLLECT* (Hooft, 1998 ▶); cell refinement: *SCALEPACK* (Otwinowski & Minor, 1997 ▶); data reduction: *DENZO* (Otwinowski & Minor, 1997 ▶) and *SCALEPACK*; program(s) used to solve structure: *SHELXS97* (Sheldrick, 2008 ▶); program(s) used to refine structure: *SHELXL97* (Sheldrick, 2008 ▶); molecular graphics: *Mercury* (Macrae *et al.*, 2008 ▶); software used to prepare material for publication: *enCIFer* (Allen *et al.*, 2004 ▶).

## Supplementary Material

Crystal structure: contains datablock(s) New_Global_Publ_Block, I. DOI: 10.1107/S1600536814009672/gk2610sup1.cif

Structure factors: contains datablock(s) I. DOI: 10.1107/S1600536814009672/gk2610Isup2.hkl

CCDC reference: 1000182

Additional supporting information:  crystallographic information; 3D view; checkCIF report

## Figures and Tables

**Table 1:** Hydrogen-bond geometry (Å, °)

*D*—H⋯*A*	*D*—H	H⋯*A*	*D*⋯*A*	*D*—H⋯*A*
O1—H1*A*⋯O11^i^	0.88 (4)	1.93 (4)	2.798 (3)	166 (3)
O11—H11*A*⋯O7	0.92 (3)	1.81 (3)	2.574 (2)	139 (3)

Symmetry code: (i) 
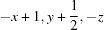
.

**Fig. 1.**
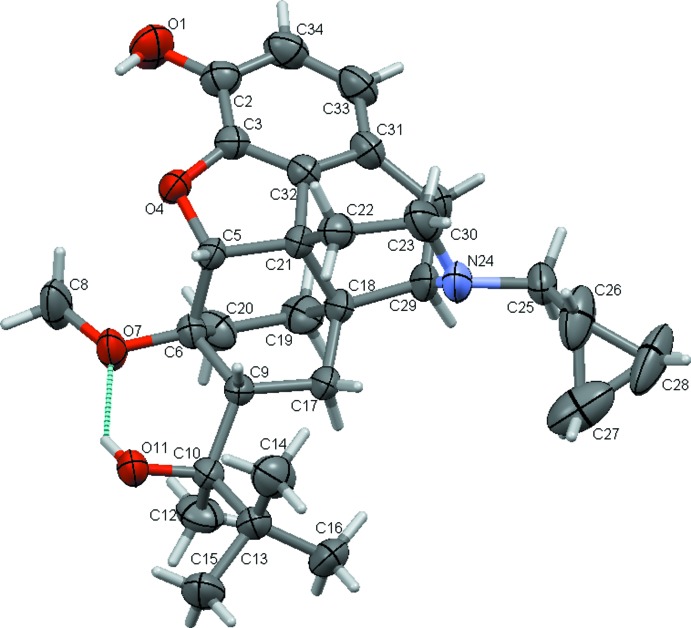
Molecular structure and atom numbering scheme for buprenorphine. Displacement ellipsoids are shown at the 50% probability level. The minor position of the disordered part has been omitted for clarity.

**Fig. 2.**
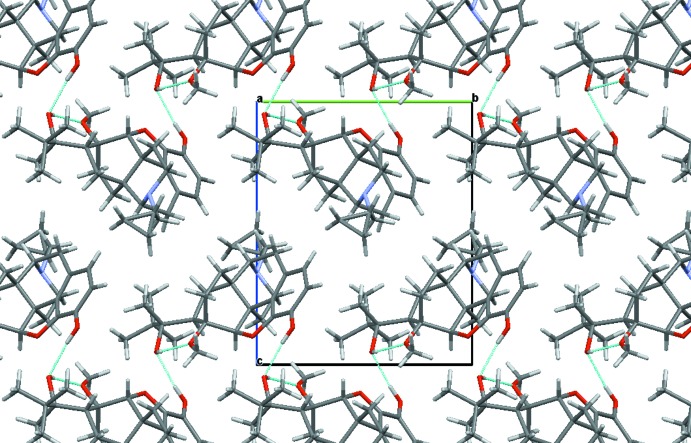
Crystal packing diagram - view along the a axis. Hydrogen bonds are shown as blue lines.

## supplementary crystallographic information

### 1. Comment

Buprenorphine is a semisynthetic opioid (Weinberg *et al.*, 1988) that is used as a pain killer. The molecule has clearly defined hydrophilic and hydrophobic parts. In the latter part, static disorder for cyclopropylmethyl group is observed with the occupation of 0.612 (8) and 0.388 (8) for the two disordered sites. The minor component of the disordered part adopts a conformation that is similar to the one observed for buprenorphine hydrochloride salt (Flippen-Anderson *et al.*, 1994; Kratochvil *et al.*, 1994). In the major component the C29-N24-C25-C26 torsion angle equals to -155.4 (4)° whereas in the minor component the corresponding C29-N24-C25A-C26A angle is -72.2 (7)°. This disorder may result from a relatively loose packing of the crystal (Kitaigorodskii packing cooeficient of 0.65 (Kitajgorodskij, 1973)) that allows for some flexibility in the hydrophobic parts of the molecule.

### 2. Experimental

Suspension of 29.6 mg of buprenorphine in 200 ml of ethyl acetate was stirred at 25 °C for 14 days. After that time the liquid was separated from the solid and left for evaporation at room temperature. After several days colorless crystals (m.p. 492.5 K) appeared that were used for diffraction studies.

### 3. Refinement

All C-bound H-atoms were included in the geometrically determined positions with *U*_iso_=1.2 *U*_eq_(C). H atoms from the OH groups were located on a Fourier difference map and refined isotropically. In the absence of significant anomalous scattering effects, Friedel pairs were merged. The absolute configuration is known from the synthetic route. The cyclopropylmethyl group is disordered over two positions. To properly model the disordered fragment restrains were imposed on some bond lengths and anisotropic thermal parameters [DFIX, SADI and SIMU commands in SHELXL-97 (Sheldrick, 2008)].

### Crystal data

**Table:**

C_29_H_41_NO_4_	*F*(000) = 508
*M**_r_* = 467.63	*D*_x_ = 1.192 Mg m^−^^3^
Monoclinic, *P*2_1_	Melting point: 492.15 K
Hall symbol: P 2yb	Mo *K*α radiation, λ = 0.71073 Å
*a* = 9.8154 (6) Å	Cell parameters from 6648 reflections
*b* = 10.4283 (9) Å	θ = 1.0–32.6°
*c* = 13.4508 (9) Å	µ = 0.08 mm^−^^1^
β = 108.796 (5)°	*T* = 296 K
*V* = 1303.37 (16) Å^3^	Block, colourless
*Z* = 2	0.45 × 0.45 × 0.25 mm

### Data collection

**Table:**

Bruker KappaCCD diffractometer	4312 reflections with *I* > 2σ(*I*)
Radiation source: fine-focus sealed tube	*R*_int_ = 0.026
Horizonally mounted graphite crystal monochromator	θ_max_ = 32.6°, θ_min_ = 3.8°
Detector resolution: 9 pixels mm^-1^	*h* = −14→14
CCD scans	*k* = −15→15
14483 measured reflections	*l* = −20→20
4886 independent reflections	

### Refinement

**Table:**

Refinement on *F*^2^	Primary atom site location: structure-invariant direct methods
Least-squares matrix: full	Secondary atom site location: difference Fourier map
*R*[*F*^2^ > 2σ(*F*^2^)] = 0.052	Hydrogen site location: inferred from neighbouring sites
*wR*(*F*^2^) = 0.146	H atoms treated by a mixture of independent and constrained refinement
*S* = 1.04	*w* = 1/[σ^2^(*F*_o_^2^) + (0.0762*P*)^2^ + 0.1599*P*] where *P* = (*F*_o_^2^ + 2*F*_c_^2^)/3
4886 reflections	(Δ/σ)_max_ = 0.007
352 parameters	Δρ_max_ = 0.32 e Å^−^^3^
112 restraints	Δρ_min_ = −0.31 e Å^−^^3^

### Special details

**Table:**

Geometry. All e.s.d.'s (except the e.s.d. in the dihedral angle between two l.s. planes) are estimated using the full covariance matrix. The cell e.s.d.'s are taken into account individually in the estimation of e.s.d.'s in distances, angles and torsion angles; correlations between e.s.d.'s in cell parameters are only used when they are defined by crystal symmetry. An approximate (isotropic) treatment of cell e.s.d.'s is used for estimating e.s.d.'s involving l.s. planes.
Refinement. Refinement of *F*^2^ against ALL reflections. The weighted *R*-factor *wR* and goodness of fit *S* are based on *F*^2^, conventional *R*-factors *R* are based on *F*, with *F* set to zero for negative *F*^2^. The threshold expression of *F*^2^ > σ(*F*^2^) is used only for calculating *R*-factors(gt) *etc*. and is not relevant to the choice of reflections for refinement. *R*-factors based on *F*^2^ are statistically about twice as large as those based on *F*, and *R*- factors based on ALL data will be even larger.

### Fractional atomic coordinates and isotropic or equivalent isotropic displacement parameters (Å^2^)

**Table:**

	*x*	*y*	*z*	*U*_iso_*/*U*_eq_	Occ. (<1)
O1	0.79854 (19)	0.6680 (2)	0.14153 (17)	0.0696 (5)	
H1A	0.754 (4)	0.621 (4)	0.086 (3)	0.078 (10)*	
C2	0.7100 (2)	0.6599 (2)	0.20083 (17)	0.0492 (4)	
C3	0.59432 (19)	0.57569 (18)	0.18247 (14)	0.0408 (3)	
O4	0.55939 (15)	0.48055 (14)	0.10755 (10)	0.0442 (3)	
C5	0.44475 (17)	0.40252 (17)	0.12545 (12)	0.0352 (3)	
H5A	0.3618	0.4015	0.0609	0.042*	
C6	0.48701 (18)	0.26326 (17)	0.15977 (13)	0.0378 (3)	
O7	0.51953 (16)	0.19164 (15)	0.07950 (13)	0.0520 (4)	
C8	0.6421 (3)	0.2234 (3)	0.0510 (3)	0.0751 (9)	
H8A	0.6489	0.1664	−0.0032	0.113*	
H8B	0.7270	0.2153	0.1112	0.113*	
H8C	0.6338	0.3101	0.0258	0.113*	
C9	0.34273 (18)	0.20874 (16)	0.17199 (13)	0.0355 (3)	
H9A	0.2657	0.2508	0.1163	0.043*	
C10	0.3121 (2)	0.06174 (16)	0.15353 (14)	0.0410 (3)	
O11	0.30844 (16)	0.03344 (14)	0.04772 (11)	0.0463 (3)	
H11A	0.401 (3)	0.054 (3)	0.050 (2)	0.056 (7)*	
C12	0.4294 (3)	−0.0206 (2)	0.2276 (2)	0.0634 (6)	
H12A	0.5217	0.0071	0.2256	0.095*	
H12B	0.4145	−0.1087	0.2060	0.095*	
H12C	0.4259	−0.0123	0.2978	0.095*	
C13	0.1567 (3)	0.0210 (2)	0.15323 (18)	0.0512 (4)	
C14	0.0423 (3)	0.1179 (3)	0.0930 (3)	0.0660 (7)	
H14A	−0.0506	0.0906	0.0941	0.099*	
H14B	0.0418	0.1233	0.0216	0.099*	
H14C	0.0641	0.2006	0.1256	0.099*	
C15	0.1171 (4)	−0.1091 (3)	0.0959 (3)	0.0722 (7)	
H15A	0.0227	−0.1344	0.0951	0.108*	
H15B	0.1858	−0.1729	0.1319	0.108*	
H15C	0.1181	−0.1006	0.0251	0.108*	
C16	0.1457 (4)	0.0033 (3)	0.2641 (2)	0.0775 (9)	
H16A	0.0492	−0.0211	0.2587	0.116*	
H16B	0.1696	0.0825	0.3023	0.116*	
H16C	0.2114	−0.0624	0.3005	0.116*	
C17	0.3285 (3)	0.26244 (19)	0.27611 (15)	0.0467 (4)	
H17A	0.2287	0.2835	0.2661	0.056*	
H17B	0.3588	0.1978	0.3307	0.056*	
C18	0.4216 (2)	0.38261 (19)	0.30967 (13)	0.0449 (4)	
C19	0.5770 (3)	0.3344 (2)	0.35075 (16)	0.0557 (5)	
H19A	0.5886	0.2774	0.4099	0.067*	
H19B	0.6420	0.4063	0.3745	0.067*	
C20	0.6138 (2)	0.2624 (2)	0.26239 (17)	0.0531 (5)	
H20A	0.6965	0.3024	0.2507	0.064*	
H20B	0.6392	0.1744	0.2840	0.064*	
C21	0.40045 (19)	0.46967 (16)	0.21299 (12)	0.0366 (3)	
C22	0.2464 (2)	0.5222 (2)	0.17321 (18)	0.0498 (4)	
H22A	0.2390	0.5842	0.1180	0.060*	
H22B	0.1805	0.4524	0.1433	0.060*	
C23	0.2020 (3)	0.5858 (3)	0.2599 (3)	0.0722 (8)	
H23A	0.2542	0.6656	0.2803	0.087*	
H23B	0.1000	0.6053	0.2342	0.087*	
N24	0.2327 (3)	0.5006 (2)	0.3518 (2)	0.0752 (7)	
C25	0.1960 (6)	0.5433 (5)	0.4459 (4)	0.0546 (11)	0.612 (8)
H25A	0.2198	0.6332	0.4601	0.065*	0.612 (8)
H25B	0.2495	0.4937	0.5070	0.065*	0.612 (8)
C25A	0.1310 (9)	0.5706 (7)	0.3988 (7)	0.0579 (17)	0.388 (8)
H25C	0.1742	0.6493	0.4331	0.069*	0.388 (8)
H25D	0.0407	0.5908	0.3450	0.069*	0.388 (8)
C26	0.0370 (5)	0.5228 (7)	0.4219 (4)	0.0817 (19)	0.612 (8)
H26	−0.0280	0.5633	0.3586	0.098*	0.612 (8)
C27	−0.0006 (12)	0.3835 (9)	0.4492 (6)	0.101 (3)	0.612 (8)
H27A	−0.0864	0.3432	0.4024	0.122*	0.612 (8)
H27B	0.0784	0.3255	0.4820	0.122*	0.612 (8)
C28	−0.0193 (12)	0.4978 (15)	0.5104 (10)	0.111 (4)	0.612 (8)
H28A	−0.1169	0.5251	0.5018	0.133*	0.612 (8)
H28B	0.0473	0.5075	0.5811	0.133*	0.612 (8)
C26A	0.1090 (8)	0.4783 (8)	0.4756 (5)	0.068 (2)	0.388 (8)
H26A	0.1901	0.4331	0.5253	0.082*	0.388 (8)
C27A	−0.0235 (18)	0.407 (2)	0.4081 (16)	0.143 (6)	0.388 (8)
H27C	−0.0687	0.4359	0.3366	0.171*	0.388 (8)
H27D	−0.0275	0.3148	0.4182	0.171*	0.388 (8)
C28A	−0.031 (2)	0.492 (3)	0.495 (2)	0.129 (7)	0.388 (8)
H28C	−0.0410	0.4516	0.5570	0.155*	0.388 (8)
H28D	−0.0822	0.5725	0.4755	0.155*	0.388 (8)
C29	0.3847 (3)	0.4674 (2)	0.39174 (18)	0.0652 (7)	
H29A	0.3989	0.4147	0.4546	0.078*	
C30	0.4872 (4)	0.5871 (3)	0.4255 (2)	0.0727 (8)	
H30A	0.4337	0.6566	0.4434	0.087*	
H30B	0.5656	0.5651	0.4884	0.087*	
C31	0.5502 (3)	0.6346 (2)	0.34350 (17)	0.0512 (4)	
C32	0.51126 (19)	0.57300 (18)	0.24722 (14)	0.0407 (3)	
C33	0.6662 (3)	0.7197 (2)	0.36339 (19)	0.0595 (5)	
H33A	0.6944	0.7668	0.4254	0.071*	
C34	0.7396 (2)	0.7347 (2)	0.2915 (2)	0.0567 (5)	
H34A	0.8112	0.7968	0.3042	0.068*	

### Atomic displacement parameters (Å^2^)

**Table:**

	*U*^11^	*U*^22^	*U*^33^	*U*^12^	*U*^13^	*U*^23^
O1	0.0602 (9)	0.0785 (13)	0.0795 (12)	−0.0284 (9)	0.0357 (9)	−0.0159 (10)
C2	0.0464 (9)	0.0461 (10)	0.0558 (10)	−0.0073 (8)	0.0174 (8)	−0.0025 (8)
C3	0.0458 (8)	0.0371 (8)	0.0408 (7)	−0.0032 (7)	0.0158 (6)	−0.0005 (6)
O4	0.0529 (7)	0.0451 (7)	0.0428 (6)	−0.0126 (6)	0.0267 (5)	−0.0058 (5)
C5	0.0413 (7)	0.0379 (7)	0.0308 (6)	−0.0052 (6)	0.0177 (5)	−0.0017 (6)
C6	0.0422 (7)	0.0368 (7)	0.0390 (7)	−0.0003 (6)	0.0197 (6)	−0.0058 (6)
O7	0.0526 (7)	0.0521 (8)	0.0643 (8)	−0.0096 (6)	0.0368 (6)	−0.0220 (7)
C8	0.0686 (13)	0.0789 (17)	0.103 (2)	−0.0219 (14)	0.0626 (14)	−0.0399 (16)
C9	0.0454 (8)	0.0309 (6)	0.0366 (7)	0.0013 (6)	0.0219 (6)	−0.0002 (6)
C10	0.0550 (9)	0.0303 (7)	0.0450 (8)	0.0014 (6)	0.0264 (7)	0.0003 (6)
O11	0.0568 (7)	0.0429 (7)	0.0489 (6)	−0.0055 (6)	0.0304 (6)	−0.0113 (5)
C12	0.0773 (15)	0.0390 (10)	0.0713 (14)	0.0109 (10)	0.0204 (12)	0.0095 (10)
C13	0.0661 (11)	0.0370 (8)	0.0646 (11)	−0.0087 (8)	0.0406 (10)	−0.0049 (8)
C14	0.0492 (11)	0.0595 (14)	0.0966 (18)	−0.0044 (10)	0.0339 (12)	−0.0078 (13)
C15	0.0888 (18)	0.0478 (12)	0.097 (2)	−0.0240 (12)	0.0537 (16)	−0.0190 (13)
C16	0.117 (2)	0.0613 (15)	0.0815 (16)	−0.0256 (16)	0.0692 (17)	−0.0056 (13)
C17	0.0724 (12)	0.0366 (8)	0.0426 (8)	−0.0043 (8)	0.0348 (8)	−0.0019 (7)
C18	0.0703 (11)	0.0386 (8)	0.0337 (7)	−0.0049 (8)	0.0277 (7)	−0.0026 (6)
C19	0.0773 (14)	0.0489 (10)	0.0340 (8)	0.0003 (10)	0.0082 (9)	0.0052 (8)
C20	0.0517 (10)	0.0477 (10)	0.0543 (10)	0.0058 (8)	0.0093 (8)	−0.0023 (9)
C21	0.0461 (8)	0.0340 (7)	0.0359 (7)	−0.0009 (6)	0.0216 (6)	−0.0008 (6)
C22	0.0485 (9)	0.0411 (9)	0.0671 (12)	0.0013 (8)	0.0289 (9)	0.0010 (8)
C23	0.0728 (14)	0.0496 (12)	0.117 (2)	−0.0031 (11)	0.0626 (15)	−0.0192 (14)
N24	0.1076 (17)	0.0555 (11)	0.0982 (16)	−0.0207 (12)	0.0827 (15)	−0.0271 (11)
C25	0.072 (3)	0.0528 (19)	0.052 (2)	0.0074 (19)	0.038 (2)	−0.0066 (17)
C25A	0.068 (4)	0.056 (3)	0.060 (4)	0.022 (3)	0.034 (3)	0.003 (3)
C26	0.065 (3)	0.127 (5)	0.064 (2)	0.040 (3)	0.035 (2)	0.012 (3)
C27	0.074 (4)	0.132 (7)	0.089 (5)	−0.023 (4)	0.014 (3)	0.043 (5)
C28	0.094 (5)	0.174 (9)	0.095 (5)	0.058 (6)	0.074 (5)	0.054 (5)
C26A	0.074 (4)	0.085 (5)	0.063 (4)	0.029 (4)	0.047 (3)	0.020 (4)
C27A	0.082 (8)	0.177 (14)	0.174 (14)	−0.044 (9)	0.048 (10)	−0.034 (12)
C28A	0.099 (10)	0.180 (15)	0.119 (12)	0.023 (11)	0.051 (8)	0.036 (12)
C29	0.114 (2)	0.0501 (12)	0.0533 (10)	−0.0199 (13)	0.0572 (13)	−0.0161 (10)
C30	0.115 (2)	0.0607 (14)	0.0589 (12)	−0.0255 (15)	0.0515 (13)	−0.0260 (12)
C31	0.0679 (12)	0.0413 (9)	0.0486 (10)	−0.0064 (9)	0.0245 (9)	−0.0119 (8)
C32	0.0480 (8)	0.0356 (8)	0.0402 (7)	−0.0018 (7)	0.0166 (6)	−0.0023 (6)
C33	0.0744 (14)	0.0455 (10)	0.0571 (11)	−0.0099 (10)	0.0190 (10)	−0.0178 (9)
C34	0.0542 (10)	0.0452 (10)	0.0675 (13)	−0.0122 (9)	0.0154 (9)	−0.0108 (10)

### Geometric parameters (Å, º)

**Table:**

O1—C2	1.358 (3)	C17—H17B	0.9700
O1—H1A	0.88 (4)	C18—C19	1.530 (3)
C2—C3	1.393 (3)	C18—C21	1.544 (2)
C2—C34	1.397 (3)	C18—C29	1.545 (3)
C3—C32	1.372 (2)	C19—C20	1.544 (3)
C3—O4	1.377 (2)	C19—H19A	0.9700
O4—C5	1.470 (2)	C19—H19B	0.9700
C5—C6	1.540 (2)	C20—H20A	0.9700
C5—C21	1.548 (2)	C20—H20B	0.9700
C5—H5A	0.9800	C21—C32	1.494 (2)
C6—O7	1.431 (2)	C21—C22	1.533 (3)
C6—C20	1.532 (3)	C22—C23	1.522 (3)
C6—C9	1.583 (2)	C22—H22A	0.9700
O7—C8	1.415 (3)	C22—H22B	0.9700
C8—H8A	0.9600	C23—N24	1.472 (4)
C8—H8B	0.9600	C23—H23A	0.9700
C8—H8C	0.9600	C23—H23B	0.9700
C9—C17	1.556 (2)	N24—C29	1.456 (4)
C9—C10	1.567 (2)	N24—C25	1.492 (4)
C9—H9A	0.9800	C25—C26	1.504 (7)
C10—O11	1.443 (2)	C25—H25A	0.9700
C10—C12	1.522 (3)	C25—H25B	0.9700
C10—C13	1.582 (3)	C26—C28	1.487 (9)
O11—H11A	0.92 (3)	C26—C27	1.572 (9)
C12—H12A	0.9600	C26—H26	0.9800
C12—H12B	0.9600	C27—C28	1.493 (10)
C12—H12C	0.9600	C27—H27A	0.9700
C13—C14	1.534 (4)	C27—H27B	0.9700
C13—C16	1.541 (3)	C28—H28A	0.9700
C13—C15	1.546 (3)	C28—H28B	0.9700
C14—H14A	0.9600	C29—C30	1.576 (4)
C14—H14B	0.9600	C29—H29A	0.9800
C14—H14C	0.9600	C30—C31	1.511 (3)
C15—H15A	0.9600	C30—H30A	0.9700
C15—H15B	0.9600	C30—H30B	0.9700
C15—H15C	0.9600	C31—C32	1.384 (3)
C16—H16A	0.9600	C31—C33	1.400 (3)
C16—H16B	0.9600	C33—C34	1.388 (4)
C16—H16C	0.9600	C33—H33A	0.9300
C17—C18	1.531 (3)	C34—H34A	0.9300
C17—H17A	0.9700		
			
C2—O1—H1A	103 (2)	C18—C19—H19A	109.8
O1—C2—C3	125.0 (2)	C20—C19—H19A	109.8
O1—C2—C34	119.08 (19)	C18—C19—H19B	109.8
C3—C2—C34	115.77 (19)	C20—C19—H19B	109.8
C32—C3—O4	113.03 (15)	H19A—C19—H19B	108.2
C32—C3—C2	121.17 (17)	C6—C20—C19	111.52 (17)
O4—C3—C2	125.50 (17)	C6—C20—H20A	109.3
C3—O4—C5	107.60 (12)	C19—C20—H20A	109.3
O4—C5—C6	115.14 (14)	C6—C20—H20B	109.3
O4—C5—C21	106.95 (13)	C19—C20—H20B	109.3
C6—C5—C21	108.25 (12)	H20A—C20—H20B	108.0
O4—C5—H5A	108.8	C32—C21—C22	112.84 (15)
C6—C5—H5A	108.8	C32—C21—C18	106.10 (14)
C21—C5—H5A	108.8	C22—C21—C18	110.72 (15)
O7—C6—C20	111.26 (15)	C32—C21—C5	101.87 (13)
O7—C6—C5	111.72 (14)	C22—C21—C5	112.53 (15)
C20—C6—C5	109.76 (15)	C18—C21—C5	112.33 (14)
O7—C6—C9	108.39 (13)	C23—C22—C21	112.4 (2)
C20—C6—C9	113.49 (15)	C23—C22—H22A	109.1
C5—C6—C9	101.90 (13)	C21—C22—H22A	109.1
C8—O7—C6	119.87 (16)	C23—C22—H22B	109.1
O7—C8—H8A	109.5	C21—C22—H22B	109.1
O7—C8—H8B	109.5	H22A—C22—H22B	107.8
H8A—C8—H8B	109.5	N24—C23—C22	110.4 (2)
O7—C8—H8C	109.5	N24—C23—H23A	109.6
H8A—C8—H8C	109.5	C22—C23—H23A	109.6
H8B—C8—H8C	109.5	N24—C23—H23B	109.6
C17—C9—C10	115.25 (14)	C22—C23—H23B	109.6
C17—C9—C6	107.89 (14)	H23A—C23—H23B	108.1
C10—C9—C6	117.87 (13)	C29—N24—C23	111.19 (18)
C17—C9—H9A	104.8	C29—N24—C25	104.9 (3)
C10—C9—H9A	104.8	C23—N24—C25	119.5 (3)
C6—C9—H9A	104.8	C29—N24—C25A	133.6 (4)
O11—C10—C12	107.66 (16)	C23—N24—C25A	94.3 (4)
O11—C10—C9	107.40 (14)	N24—C25—C26	107.0 (4)
C12—C10—C9	112.49 (17)	N24—C25—H25A	110.3
O11—C10—C13	103.00 (16)	C26—C25—H25A	110.3
C12—C10—C13	112.04 (18)	N24—C25—H25B	110.3
C9—C10—C13	113.52 (14)	C26—C25—H25B	110.3
C10—O11—H11A	101.6 (16)	H25A—C25—H25B	108.6
C10—C12—H12A	109.5	C28—C26—C25	118.7 (7)
C10—C12—H12B	109.5	C28—C26—C27	58.4 (5)
H12A—C12—H12B	109.5	C25—C26—C27	112.8 (6)
C10—C12—H12C	109.5	C28—C26—H26	117.7
H12A—C12—H12C	109.5	C25—C26—H26	117.7
H12B—C12—H12C	109.5	C27—C26—H26	117.7
C14—C13—C16	108.8 (2)	C28—C27—C26	58.0 (5)
C14—C13—C15	106.9 (2)	C28—C27—H27A	118.0
C16—C13—C15	107.1 (2)	C26—C27—H27A	118.0
C14—C13—C10	111.42 (17)	C28—C27—H27B	118.0
C16—C13—C10	113.5 (2)	C26—C27—H27B	118.0
C15—C13—C10	108.93 (18)	H27A—C27—H27B	115.1
C13—C14—H14A	109.5	C26—C28—C27	63.6 (5)
C13—C14—H14B	109.5	C26—C28—H28A	117.4
H14A—C14—H14B	109.5	C27—C28—H28A	117.4
C13—C14—H14C	109.5	C26—C28—H28B	117.4
H14A—C14—H14C	109.5	C27—C28—H28B	117.4
H14B—C14—H14C	109.5	H28A—C28—H28B	114.5
C13—C15—H15A	109.5	N24—C29—C18	108.7 (2)
C13—C15—H15B	109.5	N24—C29—C30	113.6 (2)
H15A—C15—H15B	109.5	C18—C29—C30	112.59 (18)
C13—C15—H15C	109.5	N24—C29—H29A	107.2
H15A—C15—H15C	109.5	C18—C29—H29A	107.2
H15B—C15—H15C	109.5	C30—C29—H29A	107.2
C13—C16—H16A	109.5	C31—C30—C29	115.01 (17)
C13—C16—H16B	109.5	C31—C30—H30A	108.5
H16A—C16—H16B	109.5	C29—C30—H30A	108.5
C13—C16—H16C	109.5	C31—C30—H30B	108.5
H16A—C16—H16C	109.5	C29—C30—H30B	108.5
H16B—C16—H16C	109.5	H30A—C30—H30B	107.5
C18—C17—C9	109.96 (14)	C32—C31—C33	115.8 (2)
C18—C17—H17A	109.7	C32—C31—C30	118.4 (2)
C9—C17—H17A	109.7	C33—C31—C30	124.6 (2)
C18—C17—H17B	109.7	C3—C32—C31	122.89 (18)
C9—C17—H17B	109.7	C3—C32—C21	109.98 (15)
H17A—C17—H17B	108.2	C31—C32—C21	125.23 (17)
C19—C18—C17	105.59 (17)	C34—C33—C31	120.7 (2)
C19—C18—C21	110.22 (16)	C34—C33—H33A	119.6
C17—C18—C21	109.11 (14)	C31—C33—H33A	119.6
C19—C18—C29	111.45 (18)	C33—C34—C2	122.5 (2)
C17—C18—C29	115.04 (16)	C33—C34—H34A	118.8
C21—C18—C29	105.44 (16)	C2—C34—H34A	118.8
C18—C19—C20	109.58 (16)		
			
C29—N24—C25—C26	−155.4 (4)	C29—N24—C25A—C26A	−72.2 (7)

### Hydrogen-bond geometry (Å, º)

**Table:**

*D*—H···*A*	*D*—H	H···*A*	*D*···*A*	*D*—H···*A*
O1—H1*A*···O11^i^	0.88 (4)	1.93 (4)	2.798 (3)	166 (3)
O11—H11*A*···O7	0.92 (3)	1.81 (3)	2.574 (2)	139 (3)

Symmetry code: (i) −*x*+1, *y*+1/2, −*z*.

## References

[bb1] Allen, F. H., Johnson, O., Shields, G. P., Smith, B. R. & Towler, M. (2004). *J. Appl. Cryst.* **37**, 335–338.

[bb2] Flippen-Anderson, J. L., George, C., Bertha, C. M. & Rice, K. C. (1994). *Heterocycles*, **39**, 751–766.

[bb3] Hooft, R. W. W. (1998). *COLLECT* Nonius BV, Delft, The Netherlands.

[bb4] Huang, P., Kehner, G. B., Cowan, A. & Liu-Chen, L. Y. (2001). *J. Pharmacol. Exp. Ther.* **297**, 688–695. 11303059

[bb5] Kitajgorodskij, A. I. (1973). In *Molecular Crystals and Molecules* New York: Academic Press.

[bb6] Kratochvil, B., Husak, M., Bulej, P. & Jegorov, A. (1994). *Collect. Czech. Chem. Commun.* **59**, 2472–2480.

[bb7] Macrae, C. F., Bruno, I. J., Chisholm, J. A., Edgington, P. R., McCabe, P., Pidcock, E., Rodriguez-Monge, L., Taylor, R., van de Streek, J. & Wood, P. A. (2008). *J. Appl. Cryst.* **41**, 466–470.

[bb8] Otwinowski, Z. & Minor, W. (1997). *Methods in Enzymology*, Vol. 276, *Macromolecular Crystallography*, Part A, edited by C. W. Carter Jr & R. M. Sweet, pp. 307–326. New York: Academic Press.

[bb9] Sheldrick, G. M. (2008). *Acta Cryst.* A**64**, 112–122. 10.1107/S0108767307043930 18156677

[bb10] Weinberg, D. S., Inturrisi, C. E., Reidenberg, B., Moulin, D. E., Nip, T. J., Wallenstein, S., Houde, R. W. & Foley, K. M. (1988). *Clin. Pharmacol. Ther.* **44**, 335–342. 10.1038/clpt.1988.159 2458208

